# Synthetic Biology Tools for Genome and Transcriptome Engineering of Solventogenic *Clostridium*

**DOI:** 10.3389/fbioe.2020.00282

**Published:** 2020-04-16

**Authors:** Seong Woo Kwon, Kuppusamy Alagesan Paari, Alok Malaviya, Yu-Sin Jang

**Affiliations:** ^1^Department of Agricultural Chemistry and Food Science Technology, Division of Applied Life Science (BK21 Plus Program), Institute of Agriculture & Life Science (IALS), Gyeongsang National University, Jinju, South Korea; ^2^Department of Life Sciences, CHRIST (Deemed to be University), Bengaluru, India; ^3^Applied and Industrial Biotechnology Laboratory (AIBL), Department of Life Sciences, CHRIST (Deemed to be University), Bengaluru, India

**Keywords:** *Clostridium*, synthetic biology, mobile intron, CRISPR, Cas, synthetic sRNA, UTR

## Abstract

Strains of *Clostridium* genus are used for production of various value-added products including fuels and chemicals. Development of any commercially viable production process requires a combination of both strain and fermentation process development strategies. The strain development in *Clostridium* sp. could be achieved by random mutagenesis, and targeted gene alteration methods. However, strain improvement in *Clostridium* sp. by targeted gene alteration method was challenging due to the lack of efficient tools for genome and transcriptome engineering in this organism. Recently, various synthetic biology tools have been developed to facilitate the strain engineering of solventogenic *Clostridium*. In this review, we consolidated the recent advancements in toolbox development for genome and transcriptome engineering in solventogenic *Clostridium*. Here we reviewed the genome-engineering tools employing mobile group II intron, *pyrE* alleles exchange, and CRISPR/Cas9 with their application for strain development of *Clostridium* sp. Next, transcriptome engineering tools such as untranslated region (UTR) engineering and synthetic sRNA techniques were also discussed in context of *Clostridium* strain engineering. Application of any of these discussed techniques will facilitate the metabolic engineering of clostridia for development of improved strains with respect to requisite functional attributes. This might lead to the development of an economically viable butanol production process with improved titer, yield and productivity.

## Introduction

Strain improvement for production of fuels or any biobased industrial product could be achieved by employing any of the following two strategies: (i) heterologous expression of metabolic pathway genes in a non-native producers, and (ii) improvement of native producers (Arora et al., 2019; Banerjee et al., 2019; Choi et al., 2019). However, achieving titer values in heterologous host matching to those being produced by native organisms, it requires a significant effort with high chances of failure. Therefore, the strategy of improving native strains with necessary genes of the desired pathway and cofactor regeneration capability is preferred (Park et al., 2018; Rhie et al., 2019). However, this strategy of strain improvement in *Clostridium* sp. has been limited by the availability of appropriate genome engineering tools.

*Clostridium* genus comprises many industrially important strains for biorefinery applications such as cellulosic and hemicellulosic biomass degradation, carbon fixation, advanced biofuel and platform chemical production and as anti-cancer therapeutics (Jang et al., 2012; Malaviya et al., 2012; Liu J. et al., 2015; Jones et al., 2016; Staedtke et al., 2016; Noh et al., 2018; Woo et al., 2018; Xin et al., 2018; Strecker et al., 2019a). The full potential of *Clostridium* genus for biorefinery applications could only be realized by advancement in the synthetic biology toolkits for strain improvement. During the last decade, tremendous progresses have been made in the development of genome engineering toolkit for strain engineering of *Clostridium* species. Development of genetic tools in *Clostridium* have been well reviewed by various research groups (Pyne et al., 2014; Liu Y. J. et al., 2015; Minton et al., 2016; Moon et al., 2016; Joseph et al., 2018; Kuehne et al., 2019; McAllister and Sorg, 2019; Wen et al., 2019b, c). Most of these reports are focused on couple of tools with an explanation in depth.

In this work, we have reviewed overall recent toolbox for genome and transcriptome engineering in solventogenic *Clostridium*, which could be used to develop improved clostridia strains, for production of sustainable and commercially viable industrial scale products. Brief features of the synthetic toolbox are summarized in Table 1. Consolidated information in this review dealing with strain improvement tools for *Clostridium* will aid the scientific and industrial sector to select the appropriate tools for strain improvement.

**TABLE 1:** Summary of synthetic biology tools and strategies applied for genome and transcriptome engineering of solventogenic *Clostridium*.

**Categories**	**Tools and strategy**	**Brief description**	**Selection guide**	**References**
Genome engineering	Mobile group II Intron	• Site-directed disruptions based on retrohoming of mobile group II introns • Insertion of intron into target site • Plasmid based • Ribonucleoprotein complex formation • Retrotransposition-activated selection marker (RAM) to help in selection	• Knockout • Knockdown	Chen et al., 2005, 2007; Shao et al., 2007; Heap et al., 2007; Baban et al., 2013; Jang et al., 2012, 2014; Pyne et al., 2014; Liu Y. J. et al., 2015; Liu et al., 2016; Xu et al., 2015; Meaney et al., 2015, 2016; Lawson et al., 2016
	*pyrE* allele exchange	• Works on the principle of deactivating an easily screenable gene (*pyrE*) • Complementing the mutant strain with a heterologous version of *pyrE* gene as a counter selective marker	• Knockout • Insertion • Exchange	Tripathi et al., 2010; Heap et al., 2012; Ng et al., 2013; Bankar et al., 2015; Zhang N. et al., 2015; Croux et al., 2016; Ehsaan et al., 2016a;
	CRISPR/Cas	• RNA-guided target specific DNA cleavage system • Originated from bacterial adaptive immune system • Needs single guide RNA (sgRNA), Cas endonuclease, and homologous arms for recombination	• Knockout • Knockdown	Xu et al., 2015, 2017; Nagaraju et al., 2016; Bruder et al., 2016; Li et al., 2016; Pyne et al., 2016; Wang Y. et al., 2017, Wang et al., 2018
	Phage serine integrase-mediated genome engineering	• Use two heterologous phage attachment/integration systems • Dual Integrase Cassette Exchange (DICE) strategy • Needs CRISPR/Cas9 assistance	• Knockout • Insertion	Huang et al., 2019
Transcriptome engineering	Synthetic regulatory RNA (sRNA)	• Knockdown tool based on synthetically designed sRNA • Complementarily binds to target mRNAs and block translation	• Knockdown • Overexpression (by repressor knockdown)	Cho and Lee, 2017
	Untranslated regions (UTR) engineering	• UTR modulation • Better mRNA stability by addition of small stem loop structure in the 5’-UTR	• Knockdown • Overexpression (by repressor knockdown)	Lee et al., 2016
	CRISPRi	• Knockdown tool using catalytically inactivated effector dCas9 proteins	• Knockdown • Overexpression (by repressor knockdown)	Bruder et al., 2016; Li et al., 2016; Wang et al., 2016b; Wen et al., 2017; Woolston et al., 2018; Muh et al., 2019

## Mobile Group Ii Intron Based Gene-Knockout

**FIGURE 1:**
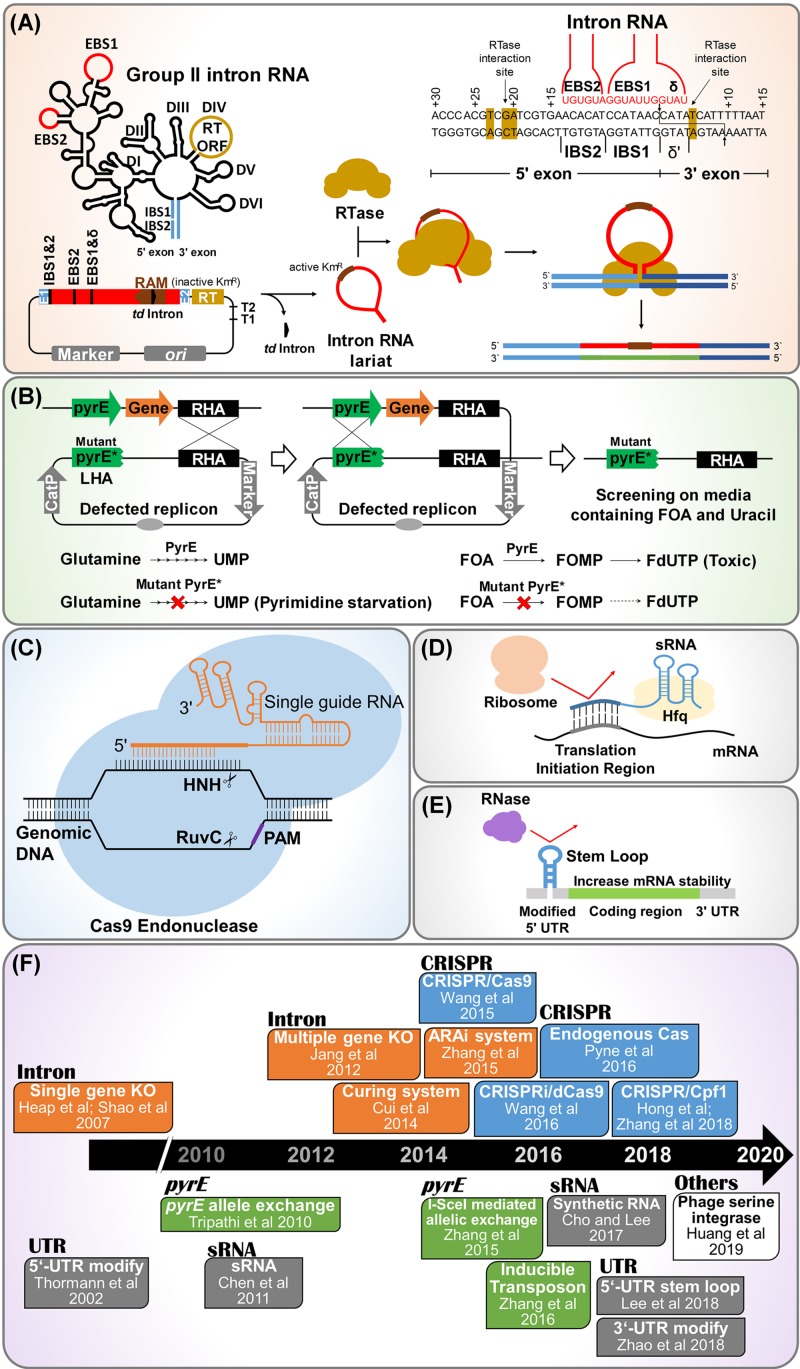
Synthetic biology tools developed for genome and transcriptome engineering of solventogenic *Clostridium*. **(A)** Mobile group II intron-based genome engineering. Also known as ClosTron in context of *Clostridium* sp. In this technology, site directed gene disruption is achieved by insertion of the mobile group II intron into the target locus of chromosome. Abbreviations: RAM, retrotranscription-activated marker (typically kanamycin resistant marker containing self-splicing group I intron, phage T4 *td* intron); RTase, reverse transcriptase; EBS, exon binding site; IBS, intron binding site. **(B)** *pyrE* based allele exchange technology for genome engineering. Here, *pyr*E encoding orotate phosphoribosyl transferase is used as counter selection marker to ensure double crossover event. The *pyrE*-mutant (PyrE^∗^) and wild type (PyrE) are resistant and sensitive to 5-fluoroorotic acid (FOA), respectively. Abbreviation: RHA, right homology arm. **(C)** CRISPR/Cas system for genome engineering. This needs single guide RNA containing crRNA and tracrRNA, Cas endonuclease, and homologous arm for recombination. Abbreviation: PAM, protospacer-adjacent motif. **(D)** Synthetic regulatory RNA (sRNA) based knockdown strategy. sRNA are having regulatory role in gene expression, mediated by chaperon Hfq. sRNA binds to complimentary mRNA sequences, prohibiting ribosome clamping at ribosome binding site located in translation initiation region. **(E)** 5′-UTR engineering for regulation of gene expression. The insertion of a small stem loop structure in the 5′-UTR increases the mRNA stability by blocking RNase, resulting in a high gene expression. **(F)** Timeline of notable events in the development of synthetic biology tools for genome and transcriptome engineering of solventogenic *Clostridium.*

Mobile group II intron technology is also known as “ClosTron” when applied in context of *Clostridium* genus. In this method a gene is disrupted by inserting the mobile intron into a target locus in the chromosome by a process termed as retrohoming, making this technology a convenient, efficient and specific method of gene disruption (Heap et al., 2007, 2010; Shao et al., 2007; Jang et al., 2012, 2014; Mohr et al., 2013; Liu Y. J. et al., 2015). Among various mobile group II introns, *Ll.LtrB* and *TeI3c/4c* have been extensively used for gene knockout in the solventogenic *Clostridium*. *Ll.LtrB* intron includes intron RNA domain and open reading frame (ORF) domain. Intron RNA domain contains splicing sites consisting of exon binding sites (EBS) 1, EBS 2, and δ (Figure 1A). The ORF domain contains genes encoding reverse transcriptase (RTase), maturase, and endonuclease (Figure 1A). *TeI3c/4c* intron has been employed to develop genome engineering tool for thermophilic *Clostridium thermocellum*, since the intron could be melted down at high temperatures (Mohr et al., 2013).

Moreover, *Ll.LtrB* intron has further been modified to include a retrotransposition-activated selection marker (RAM) (Zhong et al., 2003). RAM consists of a selection marker and is inserted into the intron. A group I intron is inserted into the marker to inactivate the marker itself. Inserted group I intron is self catalytically spliced out of mRNA in an orientation dependent manner, so that a functional marker gene can only be expressed after successful chromosomal insertion occurs (Joseph et al., 2018).

At the first stage of the clostridia gene knockout using *Ll.LtrB* intron, single gene knockouts mutant, such as *spo0A, pta, ack, ptb, buk, hbd, hydA* and *argA* variants have been constructed across the *Clostridium* genus, including *C. acetobutylicum*, *C. beijerinckii*, *C. botulinum*, and *C. difficile* (Heap et al., 2010; Dingle et al., 2011; Jang et al., 2012; Baban et al., 2013; Honicke et al., 2014; Lawson and Rainey, 2016; Liu et al., 2016). In 2012, a new method for second gene deletion was reported which could overcome the necessity of removing the plasmid used for the first gene deletion and resulted in the construction of various *C. acetobutylicum* strains, including *pta*/*buk*, *pta*/*ctfB*, *ptb*/*buk*, and triple mutant *pta*/*buk*/*ctfB* strains (Jang et al., 2012). In this technique, two genes encoding the erythromycin and chloramphenicol resistance enzymes were used as mutant selection marker and the concept of plasmid incompatibility was employed (Jang et al., 2012). In 2014, the same group reported the fourth and fifth gene deletion process for the construction of mutants *pta*/*buk*/*ctfB*/*adhE1* and *pta*/*buk*/*ctfB*/*adhE1*/*hydA* of *C. acetobutylicum* (Jang et al., 2014).

Curing and off-target manipulation remained one of the major limitations of mobile group II intron technology (Wen et al., 2019c). Curing efficiency of the plasmid containing mobile intron was enhanced by cloning *pyrF* (orotidine 5-phosphate decarboxylase) to ClosTron plasmid. The *pyrF* encodes essential enzyme of pyrimidine biosynthesis which can use 5-fluoroorotic acid (FOA) as a substrate and converts it to toxic compound and is widely used as counter selection marker (Sato et al., 2005; Tripathi et al., 2010; Heap et al., 2012). Once FOA gets converted to toxic compound by *pyrF* in the ClosTron plasmid, only cured strain could survive in the FOA added media. The cured strain can be rapidly selected by *pyrF*-based screening system, even on one plate (Cui et al., 2014).

Another problem with ClosTron is that it accidently affects and manipulates the off-target genome and cause unexpected genotypes and phenotypes (Heap et al., 2012). To overcome this, a highly regulated ClosTron system has been developed by inducing L-arabinose inducer (ARAi) to reduce off-target possibility (Zhang J. et al., 2015). To verify the impact of inducible ClosTron using ARAi system, pSY6-mspI (Cui et al., 2012) and pGZ-pyrF-cipC (Cui et al., 2014) were modified by introducing ARAi system in *C. cellulolyticum* H10 Δ*pyrF* strain. Surprisingly, it was found that the off-target manipulation frequency was decreased to 0 by inducible ClosTron ARAi system (Zhang J. et al., 2015).

## Genome Editing Using *PYRE* Alleles

Recently, allele coupled exchange (ACE) method has been developed which facilitates the insertion of complex heterologous DNA of varying size into the host genome (Ng et al., 2013; Zhang N. et al., 2015; Ehsaan et al., 2016a; Minton et al., 2016). In ACE, a counter selection marker is coupled to a desired double crossover event (Figure 1B). The counter selection marker entitles the isolation of double cross over through homologous recombination. The *pyrE* and *codA* genes are the most frequently used selectable marker in ACE Technology. The gene *codA* encodes for the enzyme cytosine deaminase while, *pyrE* encodes orotate phosphoribosyl transferase, which is a key enzyme required in the *de novo* pathway for pyrimidine biosynthesis.

In clostridia genome editing, *pyrE* allele has been primarily employed. Mutant and wild type *pyrE* allele confers resistance and sensitivity to FOA, respectively. The advantages of *pyrE* allele based recombination includes: (i) rapid insertion of heterologous DNA, (ii) double crossovers which forms the stable integration, (iii) allows large insert size, and (iv) has higher efficiency as compared to simple ClosTron and random mutagenesis (Ng et al., 2013; Ehsaan et al., 2016b; Minton et al., 2016).

The *pyrE* cassettes consists of two arms, i.e., right homology arm (RHA) and left homology arm (LHA) with the internal region comprising of *pyrE* gene (Figure 1B). A plasmid is constructed with a selectable marker (antibiotic resistance gene), origin of replication and a sequence containing ∼300-bp homologous to *pyrE* gene and a longer sequence of ∼1,200-bp homologous to the adjacent region of 3′ end of *pyrE*. Once the *pyrE* based pseudo-suicide plasmid is delivered into *Clostridium* cells, single crossover is formed through homologous recombination. Subsequently, the single crossover mutant is inoculated into the media containing FOA and uracil (Heap et al., 2012). Metabolization of FOA kills the single crossover cells carrying the active *pyrE* gene. Inactivation of *pyrE* happens only if double recombination had occurred on both 1200-bp long sequence and 300-bp short sequence and the FOA does not affect the cells obtained by such double crossovers (Ng et al., 2013). The final double crossovers are formed by ACE of shorter left homology arm of 300-bp by the second single crossover, which also leads to the excision of the plasmid (Minton et al., 2016). This technology has been found to be applicable for many species of *Clostridium* genus (Heap et al., 2012).

Butanol yield in *C. pasteurianum* has been reported to be improved by application of *pyrE* based genome editing toolkit. For this, deletion mutations were created in three genes of *C. pasteurianum*: hydrogenase (*hydA*), redox response regulator (*rex*), and glycerol dehydratase (*dhaBCE*), using plasmid pMTL-KS01. This resulted in increased availability of NADPH in cell due to depletion of 1,3-propanediol synthesis, which eventually contributed to improved butanol production (Schwarz et al., 2017). Similarly, successful expression of cellulosomal subunits in *C. acetobutylicum* has also been achieved using this method (Kovacs et al., 2013). Few other *Clostridium* species modified using ACE technology includes *C. acetobutylicum*, *C. sporogenes*, and *C. difficile* (Heap et al., 2012; Ng et al., 2013; Bankar et al., 2015; Zhang J. et al., 2015; Ehsaan et al., 2016b; Minton et al., 2016; Willson et al., 2016).

## CRISPR/Cas Based Clostridia Genome Engineering

Clustered regulatory interspaced short palindromic repeats (CRISPR) have been developed as one of the most advanced genetic engineering tools along with CRISPR-associated (Cas) protein (Doudna and Charpentier, 2014). As bacterial genome manipulation tool, CRISPR/Cas system needs single guide RNA (sgRNA), Cas endonuclease, and homologous arms for recombination (Jiang et al., 2013). The *Streptococcus pyogenes* type II CRISPR was the first CRISPR system which was exploited for genome engineering applications. Cas9 endonuclease is the basis of CRISPR based genome editing system. Cas9 recognize the protospacer adjacent motif (PAM) site (5′-NGG-3′ in *S. pyogenes*) and cleave at the 3′ end of the target gene (Mojica et al., 2009; Garneau et al., 2010; Jinek et al., 2012) (Figure 1C).

Various strains of *Clostridium* genus have been manipulated using the CRISPR/Cas9 system including *C. acetobutyricum* (Bruder et al., 2016; Li et al., 2016; Wasels et al., 2017), *C. beijerinkii* (Wang et al., 2016b), *C. autoethanogenum* (Nagaraju et al., 2016), *C. difficile* (McAllister et al., 2017; Wang et al., 2018), *C. cellulolyticum* (Xu et al., 2015, 2017), *C. pasteurianum* (Pyne et al., 2016), *C. ljungdahlii* (Huang et al., 2016), and *C. saccharoperbutylacetonicum* (Wang S. et al., 2017). However, the expression of Cas9 becomes detrimental for bacteria, including clostridia, in terms of the toxicity it causes. The mutation of 10^th^ amino acid (aspartic acid to alanine) in Cas9 inactivates it’s RuvC-like nuclease domain resulting in formation of Cas9 nickase (Cas9n), which can cleave only single-strand of DNA (Jinek et al., 2012; Nishimasu et al., 2014; Swarts and Jinek, 2018; Li et al., 2019). Cas9n have advantage in terms of overcoming the toxicity caused by expression of Cas9. Introduction of highly regulated inducible promoter for Cas9 expression is another strategy to circumvent the associated toxicity (Wang et al., 2016a; McAllister et al., 2017; Wasels et al., 2017). Nevertheless, the CRISPR/Cas9n system is still being used for clostridia genome editing (Wang et al., 2016b; Wang S. et al., 2017; Wang Y. et al., 2017; Wang et al., 2018; McAllister et al., 2017; Wasels et al., 2017).

Moreover, modified CRISPR systems like CRISPR interference (CRISPRi) and dCas9 has also been developed to knockdown of the essential genes required for host survival (Jinek et al., 2012; Qi et al., 2013; Peters et al., 2016; Zheng et al., 2019). The dCas9 has two silenced catalytic domains (D10A and H840A; RuvC-like and HNH domains, respectively) which remains bound and block the target DNA instead of cleavage. CRISPRi/dCas9 system has also been applied to develop several mutant strains of *Clostridium* sp. (Bruder et al., 2016; Wang et al., 2016a, b; Wen et al., 2017; Woolston et al., 2018; Muh et al., 2019).

Similar to Cas9, the Cpf1 from *Acidaminococcus* sp. is another protein that is used for PAM recognition in CRISPR based system. While Cas9 recognizes G-rich PAM site, the PAM recognition site for Cpf1 is T-rich (5′-TTTN-3′) (Swarts and Jinek, 2018) making it best suited for application in AT-rich organisms like *Clostridium* sp. (Zetsche et al., 2015; Yamano et al., 2016). Single CRISPR/Cpf1 system plasmid can make multiple mutants in a single application (Zetsche et al., 2017; Hong et al., 2018; Zhang et al., 2018a). CRISPR/Cpf1 system has been applied in *C. ljungdahlii*, *C. difficile*, and *C. beijerinckii* (Hong et al., 2018; Zhang et al., 2018a; Zhao et al., 2019).

Additionally, endogenous CRISPR systems have been developed in *C. pasteurianum* and *C. tyrobutyricum* to overcome the toxic effect associated with Cas9 and Cpf1 endonucleases (Pyne et al., 2016; Zhang et al., 2018b). The endogenous CRISPR system uses endonuclease encoded by the genome and can contain multiple pre-crRNAs under one promoter, facilitating multiple genome modification using a single plasmid (Luo et al., 2014; Makarova et al., 2015; Pyne et al., 2016; Zhang et al., 2018b).

## Synthetic sRNA and Untranslated Region Engineering as Potential Domains for *Clostridium* Strain Improvement

Prokaryotic small RNAs (sRNA) are short strands of ribonucleotides (about 50–500 nucleotides) which have a regulatory role in maintaining the cellular processes (Gottesman, 2004). Based on the existence of natural sRNA, synthetic small RNAs are produced to alter the gene expression of the organisms. Many such naturally occurring sRNAs have been detected and analyzed in *Clostridium* sp. (Chen et al., 2011), which leads to the development of synthetic sRNA (Na et al., 2013).

The sRNA mediated gene expression usually results in repression of the gene which complements the sRNA nucleotide sequence, mediated by a protein called Hfq (De Lay et al., 2013). Hfq is the chaperone mediated protein which stabilizes the sRNA-mRNA binding. The translation process is prevented by sRNA binding to ribosome binding site (RBS) or by masking the access to the start codon (Na et al., 2013; Yoo et al., 2013). Recently, Cho and Lee (2017) have reported the development of synthetic small regulatory RNA (sRNA) system for controlled gene expression in *C. acetobutylicum*, consisting of a target recognition site, MicC scaffold, and an RNA chaperon Hfq (Figure 1D). In this study, *C. acetobutylicum* Hfq was found to be ineffective in binding with *Escherichia coli* MicC scaffold-based synthetic sRNA, however Hfq from *E. coli* itself resulted in much enhanced knockdown efficiency. This *E. coli* MicC-Hfq sRNA system was used to knockdown *adhE1* gene expression resulting in 40% reduction in butanol production. Further, this synthetic sRNA system was used to knockdown the *pta* gene expression in PJC4BK strain, resulting in PJC4BK (pPta-Hfq^Eco^) strain with improvement of butanol titer from 14.9 to 16.9 g/l (Cho and Lee, 2017).

Untranslated regions (UTRs) are non-coding regions in the mRNA helps to regulate the gene expression. UTRs are present on both the ends of the mRNA (5′-UTR and 3′-UTR) (Figure 1E). There are sufficient reports to confirm that the 5′-UTR in *C. acetobutylicum* has the regulatory effect on the secondary structure of enzyme *adhE1*, which is involved in solvent production (Thormann et al., 2002; Scotcher et al., 2003). Lee et al. (2016) has recently found that the presence of a single stranded short 5′-UTR in the solventogenic *C. acetobutylicum* leads to decreased gene expression (Figure 1E). The insertion of a small stem loop structure in the 5′-UTR was found to increase the mRNA stability and gene expression by 4.6 folds, without any modification in the promoter or RBS (Lee et al., 2016). On the other hand, the 3′-UTR mostly harbors the terminator sequence for transcription process in mRNA (Richard and Manley, 2009). sRNA sequence containing the codons that regulates the post transcriptional and translation machinery is also attached to 3′-UTR. Most importantly 3′-UTR confer stability to the mRNA (Zhao et al., 2018). Although, there are very limited studies related to 3′-UTR regions in *Clostridium*, the presence of transcripts with long 3′-UTR is confirmed in *Clostridium* (Ralston and Papoutsakis, 2018). Although several RNAseq studies were reported in the *Clostridium*, only few studies show the data related to regulation of mRNA based on 5′- and 3′-UTRs, leaderless transcripts and non-coding RNA (Soutourina et al., 2013; Wilson et al., 2013; Sedlar et al., 2018). Further research in RNAseq and proteomics will explore the complex regulations that control mRNA stability and degradation, which will be more useful to construction synthetic toolkit.

In conclusion, many *Clostridium* sp. have potential to be utilized at industrial scale to produce value added chemicals, including butanol as fossil fuel substitute. Up to date, their true potential was underexploited due to challenges in strain improvement and unavailability of genome and transcriptome editing tools for this genus. Nevertheless, during the last decade, synthetic biology toolkits for *Clostridium* sp. have been expanded rapidly (Figure 1F). Furthermore, a recent advancement, such as phage serine integrase mediated site-specific genome engineering technique for *C. ljungdahlii* could be extended to other *Clostridium* species (Huang et al., 2019). The synthetic biology techniques that have been applied in other microorganisms may also be adopted to solventogenic clostridia in the near future: CRISPR associated site-specific insertion of transposons and base editing techniques (Ronda et al., 2015; Zhang et al., 2016; Lim and Choi, 2019; Strecker et al., 2019b). Utilization of improved clostridia strains could be a starting point for development of an industrial scale, commercially viable bio-based fuel and chemical production using *Clostridium* sp. using a consolidated bioprocessing concept (Wen et al., 2019a). Furthermore, these synthetic biology tools could be applied to another biotechnology fields such as degradation of plastics, such as polyethylene terephthalate and polyethylene.

## Author Contributions

Y-SJ and AM conceived the project. All authors analyzed the literature, compiled data, planned content, wrote the manuscript, read, and approved the final manuscript.

## Conflict of Interest

The authors declare that the research was conducted in the absence of any commercial or financial relationships that could be construed as a potential conflict of interest.

## References

[B1] Arora R. Behera S. Sharma N. K. Kumar S. (2019). Evaluating the pathway for co-fermentation of glucose and xylose for enhanced bioethanol production using flux balance analysis. *Biotechnol. Bioproc. Eng.* 24 924–933. 10.1007/s12257-019-0026-5

[B2] Baban S. T. Kuehne S. A. Barketi-Klai A. Cartman S. T. Kelly M. L. Hardie K. R. (2013). The role of flagella in *Clostridium difficile* pathogenesis: comparison between a non-epidemic and an epidemic strain. *PLoS One* 8:e73026 10.1371/journal.pone.0073026 PMC3781105 24086268

[B3] Banerjee S. Mishra G. Roy A. (2019). Metabolic engineering of bacteria for renewable bioethanol production from cellulosic biomass. *Biotechnol. Bioproc. Eng.* 24 713–733. 10.1007/s12257-019-0134-2

[B4] Bankar S. B. Jurgens G. Survase S. A. Ojamo H. Granström T. (2015). Genetic engineering of *Clostridium acetobutylicum* to enhance isopropanol-butanol-ethanol production with an integrated DNA-technology approach. *Renew. Energy* 83 1076–1083. 10.1016/j.renene.2015.05.052

[B5] Bruder M. R. Pyne M. E. Moo-Young M. Chung D. A. Chou C. P. (2016). Extending CRISPR-Cas9 technology from genome editing to transcriptional engineering in the genus *Clostridium*. *Appl. Environ. Microbiol.* 82 6109–6119. 10.1128/aem.02128-16 27496775 PMC5068152

[B6] Chen Y. Caruso L. McClane B. Fisher D. Gupta P. (2007). Disruption of a toxin gene by introduction of a foreign gene into the chromosome of *Clostridium perfringens* using targetron-induced mutagenesis. *Plasmid* 58, 182–189. 10.1016/j.plasmid.2007.04.002 17553563 PMC2034400

[B7] Chen Y. Indurthi D. C. Jones S. W. Papoutsakis E. T. (2011). Small RNAs in the genus *Clostridium*. *mBio* 2:e340-10. 10.1128/mBio.00340-10 PMC3025663 21264064

[B8] Chen Y. McClane B. A. Fisher D. J. Rood J. I. Gupta P. (2005). Construction of an alpha toxin gene knockout mutant of *Clostridium perfringens* type A by use of a mobile group II intron. *Appl. Environ. Microbiol.* 71, 7542–7547. 10.1128/AEM.71.11.7542-7547.2005 16269799 PMC1287605

[B9] Cho C. Lee S. Y. (2017). Efficient gene knockdown in *Clostridium acetobutylicum* by synthetic small regulatory RNAs. *Biotechnol. Bioeng.* 114 374–383. 10.1002/bit.26077 27531464

[B10] Choi Y. Y. Hong M.-E. Chang W. S. Sim S. J. (2019). Autotrophic biodiesel production from the thermotolerant microalga C*hlorella sorokiniana* by enhancing the carbon availability with temperature adjustment. *Biotechnol. Bioproc. Eng.* 24 223–231. 10.1007/s12257-018-0375-5

[B11] Croux C. Nguyen N. P. Lee J. Raynaud C. Saint-Prix F. Gonzalez-Pajuelo M. (2016). Construction of a restriction-less, marker-less mutant useful for functional genomic and metabolic engineering of the biofuel producer *Clostridium acetobutylicum*. *Biotechnol. Biofuels* 9:23 10.1186/s13068-016-0432-2 PMC4736252 26839586

[B12] Cui G. Z. Hong W. Zhang J. Li W. L. Feng Y. Liu Y. J. (2012). Targeted gene engineering in *Clostridium cellulolyticum* H10 without methylation. *J. Microbiol. Methods* 89 201–208. 10.1016/j.mimet.2012.02.015 22450138

[B13] Cui G. Z. Zhang J. Hong W. Xu C. Feng Y. Cui Q. (2014). Improvement of ClosTron for successive gene disruption in *Clostridium cellulolyticum* using a *pyrF*-based screening system. *Appl. Microbiol. Biotechnol.* 98 313–323. 10.1007/s00253-013-5330-y 24190496

[B14] De Lay N. Schu D. J. Gottesman S. (2013). Bacterial small RNA-based negative regulation: Hfq and its accomplices. *J. Biol. Chem.* 288 7996–8003. 10.1074/jbc.r112.441386 23362267 PMC3605619

[B15] Dingle T. C. Mulvey G. L. Armstrong G. D. (2011). Mutagenic analysis of the *Clostridium difficile* flagellar proteins, FliC and FliD, and their contribution to virulence in hamsters. *Infect. Immun.* 79 4061–4067. 10.1128/iai.05305-11 21788384 PMC3187235

[B16] Doudna J. A. Charpentier E. (2014). Genome editing. The new frontier of genome engineering with CRISPR-Cas9. *Science* 346:1258096. 10.1126/science.1258096 25430774

[B17] Ehsaan M. Kuehne S. A. Minton N. P. (2016a). “*Clostridium difficile* genome editing using pyrE alleles,” in *Clostridium Difficile*, eds Roberts A. P. Mullany P. (Berlin: Springer), 35–52. 10.1007/978-1-4939-6361-4_4 27507332

[B18] Ehsaan M. Kuit W. Zhang Y. Cartman S. T. Heap J. T. Winzer K. (2016b). Mutant generation by allelic exchange and genome resequencing of the biobutanol organism *Clostridium acetobutylicum* ATCC 824. *Biotechnol. Biofuels* 9:4. 10.1186/s13068-015-0410-0 PMC4700727 26732067

[B19] Garneau J. E. Dupuis M. E. Villion M. Romero D. A. Barrangou R. Boyaval P. (2010). The CRISPR/Cas bacterial immune system cleaves bacteriophage and plasmid DNA. *Nature* 468 67–71. 10.1038/nature09523 21048762

[B20] Gottesman S. (2004). The small RNA regulators of *Escherichia coli*: roles and mechanisms. *Annu. Rev. Microbiol.* 58 303–328. 10.1146/annurev.micro.58.030603.123841 15487940

[B21] Heap J. T. Ehsaan M. Cooksley C. M. Ng Y. K. Cartman S. T. Winzer K. (2012). Integration of DNA into bacterial chromosomes from plasmids without a counter-selection marker. *Nucleic Acids Res.* 40:e59 10.1093/nar/gkr1321 PMC3333862 22259038

[B22] Heap J. T. Kuehne S. A. Ehsaan M. Cartman S. T. Cooksley C. M. Scott J. C. (2010). The ClosTron: mutagenesis in *Clostridium* refined and streamlined. *J. Microbiol. Methods* 80 49–55. 10.1016/j.mimet.2009.10.018 19891996

[B23] Heap J. T. Pennington O. J. Cartman S. T. Carter G. P. Minton N. P. (2007). The ClosTron: a universal gene knock-out system for the genus *Clostridium*. *J. Microbiol. Methods* 70 452–464. 10.1016/j.mimet.2007.05.021 17658189

[B24] Hong W. Zhang J. Cui G. Wang L. Wang Y. (2018). Multiplexed CRISPR-Cpf1-mediated genome editing in *Clostridium difficile* toward the understanding of pathogenesis of *C. difficile* infection. *ACS Synth. Biol.* 7 1588–1600. 10.1021/acssynbio.8b00087 29863336

[B25] Honicke D. Lutke-Eversloh T. Liu Z. Lehmann D. Liebl W. Ehrenreich A. (2014). Chemostat cultivation and transcriptional analyses of *Clostridium acetobutylicum* mutants with defects in the acid and acetone biosynthetic pathways. *Appl. Microbiol. Biotechnol.* 98 9777–9794. 10.1007/s00253-014-6040-9 25280743

[B26] Huang H. Chai C. Li N. Rowe P. Minton N. P. Yang S. (2016). CRISPR/Cas9-Based efficient genome editing in *Clostridium ljungdahlii*, an autotrophic gas-fermenting bacterium. *ACS Synth. Biol.* 5 1355–1361. 10.1021/acssynbio.6b00044 27276212

[B27] Huang H. Chai C. Yang S. Jiang W. Gu Y. (2019). Phage serine integrase-mediated genome engineering for efficient expression of chemical biosynthetic pathway in gas-fermenting *Clostridium ljungdahlii*. *Metab. Eng* 52 293–302. 10.1016/j.ymben.2019.01.005 30633974

[B28] Jang Y.-S. Im J. A. Choi S. Y. Lee J. I. Lee S. Y. (2014). Metabolic engineering of *Clostridium acetobutylicum* for butyric acid production with high butyric acid selectivity. *Metab. Eng.* 23 165–174. 10.1016/j.ymben.2014.03.004 24704310

[B29] Jang Y. S. Lee J. Y. Lee J. Park J. H. Im J. A. Eom M. H. (2012). Enhanced butanol production obtained by reinforcing the direct butanol-forming route in *Clostridium acetobutylicum*. *mBio* 3:e00314-12. 10.1128/mBio.00314-12 PMC3482502 23093384

[B30] Jiang W. Bikard D. Cox D. Zhang F. Marraffini L. A. (2013). RNA-guided editing of bacterial genomes using CRISPR-Cas systems. *Nat. Biotechnol.* 31 233–239. 10.1038/nbt.2508 23360965 PMC3748948

[B31] Jinek M. Chylinski K. Fonfara I. Hauer M. Doudna J. A. Charpentier E. (2012). A programmable dual-RNA-guided DNA endonuclease in adaptive bacterial immunity. *Science* 337 816–821. 10.1126/science.1225829 22745249 PMC6286148

[B32] Jones A. J. Venkataramanan K. P. Papoutsakis T. (2016). Overexpression of two stress-responsive, small, non-coding RNAs, 6S and tmRNA, imparts butanol tolerance in *Clostridium acetobutylicum*. *FEMS Microbiol. Lett.* 363:fnw063 10.1093/femsle/fnw063 26989157

[B33] Joseph R. C. Kim N. M. Sandoval N. R. (2018). Recent developments of the synthetic biology toolkit for *Clostridium*. *Front. Microbiol.* 9:154 10.3389/fmicb.2018.00154 PMC5816073 29483900

[B34] Kovacs K. Willson B. J. Schwarz K. Heap J. T. Jackson A. Bolam D. N. (2013). Secretion and assembly of functional mini-cellulosomes from synthetic chromosomal operons in *Clostridium acetobutylicum* ATCC 824. *Biotechnol. Biofuels* 6:117 10.1186/1754-6834-6-117 PMC3765823 23962085

[B35] Kuehne S. A. Rood J. I. Lyras D. (2019). Clostridial genetics: genetic manipulation of the pathogenic clostridia. *Microbiol Spectr* 7:GPP3-0040-2018. 10.1128/microbiolspec.gpp3-0040-2018 PMC11315012 31172914

[B36] Lawson P. A. Rainey F. A. (2016). Proposal to restrict the genus *Clostridium prazmowski* to *Clostridium butyricum* and related species. *Int. J. Syst. Evol. Microbiol.* 66 1009–1016. 10.1099/ijsem.0.000824 26643615

[B37] Lawson P. A. Citron D. M. Tyrrell K. L. Finegold S. M. (2016). Reclassification of *Clostridium difficile* as *Clostridioides difficile* (Hall and O’Toole 1935) Prevot 1938. *Anaerobe* 40, 95–99. 10.1016/j.anaerobe.2016.06.008 27370902

[B38] Lee J. Jang Y. S. Papoutsakis E. T. Lee S. Y. (2016). Stable and enhanced gene expression in *Clostridium acetobutylicum* using synthetic untranslated regions with a stem-loop. *J. Biotechnol.* 230 40–43. 10.1016/j.jbiotec.2016.05.020 27188957

[B39] Li Q. Chen J. Minton N. P. Zhang Y. Wen Z. Liu J. (2016). CRISPR-based genome editing and expression control systems in *Clostridium acetobutylicum* and *Clostridium beijerinckii*. *Biotechnol. J.* 11 961–972. 27213844 10.1002/biot.201600053

[B40] Li Q. Seys F. M. Minton N. P. Yang J. Jiang Y. Jiang W. (2019). CRISPR-Cas9(D10A) nickase-assisted base editing in the solvent producer *Clostridium beijerinckii*. *Biotechnol. Bioeng.* 116 1475–1483. 10.1002/bit.26949 30739328

[B41] Lim H. Choi S. K. (2019). Programmed gRNA removal system for CRISPR-Cas9-mediated multi-round genome editing in *Bacillus subtilis*. *Front. Microbiol.* 10:1140 10.3389/fmicb.2019.01140 PMC6536666 31164882

[B42] Liu J. Guo T. Wang D. Shen X. Liu D. Niu H. (2016). Enhanced butanol production by increasing NADH and ATP levels in *Clostridium beijerinckii* NCIMB 8052 by insertional inactivation of Cbei_4110. *Appl. Microbiol. Biotechnol.* 100 4985–4996. 10.1007/s00253-016-7299-9 26830101

[B43] Liu J. Sun J. Wang F. Yu X. Ling Z. Li H. (2015). Neuroprotective effects of *Clostridium butyricum* against vascular dementia in mice via metabolic butyrate. *Biomed. Res. Int.* 2015:412946. 10.1155/2015/412946 PMC4615854 26523278

[B44] Liu Y. J. Zhang J. Cui G. Z. Cui Q. (2015). Current progress of targetron technology: development, improvement and application in metabolic engineering. *Biotechnol. J.* 10 855–865. 10.1002/biot.201400716 25735546

[B45] Luo M. L. Mullis A. S. Leenay R. T. Beisel C. L. (2014). Repurposing endogenous type I CRISPR-Cas systems for programmable gene repression. *Nucleic Acids Res.* 43 674–681. 10.1093/nar/gku971 25326321 PMC4288209

[B46] Makarova K. S. Wolf Y. I. Alkhnbashi O. S. Costa F. Shah S. A. Saunders S. J. (2015). An updated evolutionary classification of CRISPR-Cas systems. *Nat. Rev. Microbiol.* 13 722–736. 26411297 10.1038/nrmicro3569 PMC5426118

[B47] Malaviya A. Jang Y. S. Lee S. Y. (2012). Continuous butanol production with reduced byproducts formation from glycerol by a hyper producing mutant of *Clostridium pasteurianum*. *Appl. Microbiol. Biotechnol.* 93 1485–1494. 10.1007/s00253-011-3629-0 22052388

[B48] McAllister K. N. Bouillaut L. Kahn J. N. Self W. T. Sorg J. A. (2017). Using CRISPR-Cas9-mediated genome editing to generate *C. difficile* mutants defective in selenoproteins synthesis. *Sci. Rep.* 7:14672. 10.1038/s41598-017-15236-5 PMC5677094 29116155

[B49] McAllister K. N. Sorg J. A. (2019). CRISPR genome editing systems in the genus *Clostridium*: a timely advancement. *J. Bacteriol.* 201:e00 219–19. 10.1128/JB.00219-19 PMC6657597 31085694

[B50] Meaney C. A. Cartman S. T. McClure P. J. Minton N. P. (2015). Optimal spore germination in *Clostridium botulinum* ATCC 3502 requires the presence of functional copies of SleB and YpeB, but not CwlJ. *Anaerobe* 34, 86–93. 25937262 10.1016/j.anaerobe.2015.04.015

[B51] Meaney C. A. Cartman S. T. McClure P.J. Minton N. P. (2016). The role of small acid-soluble proteins (SASPs) in protection of spores of *Clostridium botulinum* against nitrous acid. *Int. J. Food Microbiol.* 216, 25–30. 10.1016/j.ijfoodmicro.2015.08.024 26386202

[B52] Minton N. P. Ehsaan M. Humphreys C. M. Little G. T. Baker J. Henstra A. M. (2016). A roadmap for gene system development in *Clostridium*. *Anaerobe* 41 104–112. 10.1016/j.anaerobe.2016.05.011 27234263 PMC5058259

[B53] Mohr G. Hong W. Zhang J. Cui G.-Z. Yang Y. Cui Q. (2013). A targetron system for gene targeting in thermophiles and its application in *Clostridium thermocellum*. *PLoS ONE* 8:e69032 10.1371/journal.pone.0069032 PMC3706431 23874856

[B54] Mojica F. J. Diez-Villasenor C. Garcia-Martinez J. Almendros C. (2009). Short motif sequences determine the targets of the prokaryotic CRISPR defence system. *Microbiology* 155 733–740. 10.1099/mic.0.023960-0 19246744

[B55] Moon H. G. Jang Y. S. Cho C. Lee J. Binkley R. Lee S. Y. (2016). One hundred years of clostridial butanol fermentation. *FEMS Microbiol Lett* 363:fnw001 10.1093/femsle/fnw001 26738754

[B56] Muh U. Pannullo A. G. Weiss D. S. Ellermeier C. D. (2019). A xylose-inducible expression system and a CRISPR interference plasmid for targeted knockdown of gene expression in *Clostridioides difficile*. *J. Bacteriol.* 201 e711–e718. 10.1128/JB.00711-18 PMC6597395 30745377

[B57] Na D. Yoo S. M. Chung H. Park H. Park J. H. Lee S. Y. (2013). Metabolic engineering of *Escherichia coli* using synthetic small regulatory RNAs. *Nat. Biotechnol.* 31 170–174. 10.1038/nbt.2461 23334451

[B58] Nagaraju S. Davies N. K. Walker D. J. Kopke M. Simpson S. D. (2016). Genome editing of *Clostridium autoethanogenum* using CRISPR/Cas9. *Biotechnol. Biofuels* 9:219. 10.1186/s13068-016-0638-3 PMC5069954 27777621

[B59] Ng Y. K. Ehsaan M. Philip S. Collery M. M. Janoir C. Collignon A. (2013). Expanding the repertoire of gene tools for precise manipulation of the *Clostridium difficile* genome: allelic exchange using *pyrE* alleles. *PLoS ONE* 8:e56051 10.1371/journal.pone.0056051 PMC3566075 23405251

[B60] Nishimasu H. Ran F. A. Hsu P. D. Konermann S. Shehata S. I. Dohmae N. (2014). Crystal structure of Cas9 in complex with guide RNA and target DNA. *Cell* 156 935–949. 10.1016/j.cell.2014.02.001 24529477 PMC4139937

[B61] Noh H. J. Woo J. E. Lee S. Y. Jang Y.-S. (2018). Metabolic engineering of *Clostridium acetobutylicum* for the production of butyl butyrate. *Appl. Microbiol. Biotechnol.* 102 8319–8327. 10.1007/s00253-018-9267-z 30076425

[B62] Park M.-R. Kim S.-K. Jeong G.-T. (2018). Biosugar production from *Gracilaria verrucosa* with sulfamic acid pretreatment and subsequent enzymatic hydrolysis. *Biotechnol. Bioproc. Eng.* 23 302–310. 10.1007/s12257-018-0090-2 27003825

[B63] Peters J. M. Colavin A. Shi H. Czarny T. L. Larson M. H. Wong S. (2016). A comprehensive, CRISPR-based functional analysis of essential genes in bacteria. *Cell* 165 1493–1506. 10.1016/j.cell.2016.05.003 27238023 PMC4894308

[B64] Pyne M. E. Bruder M. Moo-Young M. Chung D. A. Chou C. P. (2014). Technical guide for genetic advancement of underdeveloped and intractable *Clostridium*. *Biotechnol. Adv.* 32 623–641. 10.1016/j.biotechadv.2014.04.003 24768687

[B65] Pyne M. E. Bruder M. R. Moo-Young M. Chung D. A. Chou C. P. (2016). Harnessing heterologous and endogenous CRISPR-Cas machineries for efficient markerless genome editing in *Clostridium*. *Sci. Rep.* 6:25666. 10.1038/srep25666 PMC4860712 27157668

[B66] Qi L. S. Larson M. H. Gilbert L. A. Doudna J. A. Weissman J. S. Arkin A. P. (2013). Repurposing CRISPR as an RNA-guided platform for sequence-specific control of gene expression. *Cell* 152 1173–1183. 10.1016/j.cell.2013.02.022 23452860 PMC3664290

[B67] Ralston M. T. Papoutsakis E. T. (2018). RNAseq-based transcriptome assembly of *Clostridium acetobutylicum* for functional genome annotation and discovery. *AIChE J.* 64 4271–4280. 10.1002/aic.16396

[B68] Rhie M. N. Kim H. T. Jo S. Y. Chu L. L. Baritugo K.-A. Baylon M. G. (2019). Recent advances in the metabolic engineering of *Klebsiella pneumoniae*: a potential platform microorganism for biorefineries. *Biotechnol. Bioproc. Eng.* 24 48–64. 10.1007/s12257-018-0346-x

[B69] Richard P. Manley J. L. (2009). Transcription termination by nuclear RNA polymerases. *Genes Dev.* 23 1247–1269. 10.1101/gad.1792809 19487567 PMC2763537

[B70] Ronda C. Maury J. Jakociunas T. Jacobsen S. A. Germann S. M. Harrison S. J. (2015). CrEdit: CRISPR mediated multi-loci gene integration in *Saccharomyces cerevisiae*. *Microb. Cell Fact.* 14:97. 10.1186/s12934-015-0288-3 PMC4492099 26148499

[B71] Sato T. Fukui T. Atomi H. Imanaka T. (2005). Improved and versatile transformation system allowing multiple genetic manipulations of the hyperthermophilic archaeon *Thermococcus kodakaraensis*. *Appl. Environ. Microbiol.* 71 3889–3899. 10.1128/aem.71.7.3889-3899.2005 16000802 PMC1169065

[B72] Schwarz K. M. Grosse-Honebrink A. Derecka K. Rotta C. Zhang Y. Minton N. P. (2017). Towards improved butanol production through targeted genetic modification of *Clostridium pasteurianum*. *Metab. Eng.* 40 124–137. 10.1016/j.ymben.2017.01.009 28119139 PMC5367854

[B73] Scotcher M. C. Huang K. X. Harrison M. L. Rudolph F. B. Bennett G. N. (2003). Sequences affecting the regulation of solvent production in *Clostridium acetobutylicum*. *J. Ind. Microbiol. Biotechnol.* 30 414–420. 10.1007/s10295-003-0057-x 12774196

[B74] Sedlar K. Koscova P. Vasylkivska M. Branska B. Kolek J. Kupkova K. (2018). Transcription profiling of butanol producer *Clostridium beijerinckii* NRRL B-598 using RNA-Seq. *BMC Genomics* 19:415 10.1186/s12864-018-4805-8 PMC5975590 29843608

[B75] Shao L. Hu S. Yang Y. Gu Y. Chen J. Yang Y. (2007). Targeted gene disruption by use of a group II intron (targetron) vector in *Clostridium acetobutylicum*. *Cell Res.* 17 963–965. 10.1038/cr.2007.91 17971808

[B76] Soutourina O. A. Monot M. Boudry P. Saujet L. Pichon C. Sismeiro O. (2013). Genome-wide identification of regulatory RNAs in the human pathogen *Clostridium difficile*. *PLoS Genet.* 9:e1003493 10.1371/journal.pgen.1003493 PMC3649979 23675309

[B77] Staedtke V. Roberts N. J. Bai R. Y. Zhou S. (2016). *Clostridium novyi*-NT in cancer therapy. *Genes Dis.* 3 144–152. 10.1016/j.gendis.2016.01.003 30258882 PMC6150096

[B78] Strecker J. Jones S. Koopal B. Schmid-Burgk J. Zetsche B. Gao L. (2019a). Engineering of CRISPR-Cas12b for human genome editing. *Nat. Commun.* 10:212 10.1017/cbo9781316771440.007 PMC6342934 30670702

[B79] Strecker J. Ladha A. Gardner Z. Schmid-Burgk J. L. Makarova K. S. Koonin E. V. (2019b). RNA-guided DNA insertion with CRISPR-associated transposases. *Science* 365 48–53. 10.1126/science.aax9181 31171706 PMC6659118

[B80] Swarts D. C. Jinek M. (2018). *Cas9 versus Cas12a/Cpf1: Structure-Function Comparisons and Implications for Genome Editing.* Hoboken, NJ: Wiley Interdiscip. Rev. RNA, e1481. 10.1002/wrna.1481 29790280

[B81] Thormann K. Feustel L. Lorenz K. Nakotte S. Durre P. (2002). Control of butanol formation in *Clostridium acetobutylicum* by transcriptional activation. *J. Bacteriol.* 184 1966–1973. 10.1128/jb.184.7.1966-1973.2002 11889105 PMC134926

[B82] Tripathi S. A. Olson D. G. Argyros D. A. Miller B. B. Barrett T. F. Murphy D. M. (2010). Development of *pyrF*-based genetic system for targeted gene deletion in *Clostridium thermocellum* and creation of a *pta* mutant. *Appl. Environ. Microbiol.* 76 6591–6599. 10.1128/aem.01484-10 20693441 PMC2950449

[B83] Wang S. Dong S. Wang P. Tao Y. Wang Y. (2017). Genome editing in *Clostridium saccharoperbutylacetonicum* N1-4 with the CRISPR-Cas9 system. *Appl. Environ. Microbiol.* 83:e233-17. 10.1128/AEM.00233-17 PMC5411512 28258147

[B84] Wang Y. Zhang G. Zhao X. Ling J. (2017). Genome shuffling improved the nucleosides production in *Cordyceps kyushuensis*. *J. Biotechnol.* 260 42–47. 10.1016/j.jbiotec.2017.08.021 28882571

[B85] Wang S. Hong W. Dong S. Zhang Z. T. Zhang J. Wang L. (2018). Genome engineering of *Clostridium difficile* using the CRISPR-Cas9 system. *Clin. Microbiol. Infect.* 24 1095–1099. 29604353 10.1016/j.cmi.2018.03.026

[B86] Wang Y. Zhang Z. T. Seo S. O. Lynn P. Lu T. Jin Y. S. (2016a). Bacterial genome editing with CRISPR-Cas9: deletion, integration, single nucleotide modification, and desirable “clean” mutant selection in *Clostridium beijerinckii* as an example. *ACS Synth. Biol.* 5 721–732. 10.1021/acssynbio.6b00060 27115041

[B87] Wang Y. Zhang Z. T. Seo S. O. Lynn P. Lu T. Jin Y. S. (2016b). Gene transcription repression in *Clostridium beijerinckii* using CRISPR-dCas9. *Biotechnol. Bioeng.* 113 2739–2743. 10.1002/bit.26020 27240718

[B88] Wasels F. Jean-Marie J. Collas F. Lopez-Contreras A. M. Lopes Ferreira N. (2017). A two-plasmid inducible CRISPR/Cas9 genome editing tool for *Clostridium acetobutylicum*. *J. Microbiol. Methods* 140 5–11. 10.1016/j.mimet.2017.06.010 28610973

[B89] Wen Z. Ledesma-Amaro R. Lin J. Jiang Y. Yang S. (2019a). Improved n-butanol production from *Clostridium cellulovorans* by integrated metabolic and evolutionary engineering. *Appl. Environ. Microbiol* 85 e2560–e2518. 10.1128/AEM.02560-18 PMC6585503 30658972

[B90] Wen Z. Li Q. Liu J. Jin M. Yang S. (2019b). Consolidated bioprocessing for butanol production of cellulolytic clostridia: development and optimization. *Microb. Biotechnol.* 13 410–422. 10.1111/1751-7915.13478 31448546 PMC7017829

[B91] Wen Z. Lu M. Ledesma-Amaro R. Li Q. Jin M. Yang S. (2019c). Targetron technology applicable in solventogenic clostridia: revisiting 12 years’ advances. *Biotechnol. J.* 15:e1900284. 10.1002/biot.201900284 31475782

[B92] Wen Z. Minton N. P. Zhang Y. Li Q. Liu J. Jiang Y. (2017). Enhanced solvent production by metabolic engineering of a twin-clostridial consortium. *Metab. Eng.* 39 38–48. 10.1016/j.ymben.2016.10.013 27794465

[B93] Willson B. J. Kovacs K. Wilding-Steele T. Markus R. Winzer K. Minton N. P. (2016). Production of a functional cell wall-anchored minicellulosome by recombinant *Clostridium acetobutylicum* ATCC 824. *Biotechnol. Biofuels* 9:109. 10.1186/s13068-016-0526-x PMC4877998 27222664

[B94] Wilson C. M. Rodriguez M. Jr. Johnson C. M. Martin S. L. Chu T. M. Wolfinger R. D. (2013). Global transcriptome analysis of *Clostridium thermocellum* ATCC 27405 during growth on dilute acid pretreated *Populus* and switchgrass. *Biotechnol. Biofuels* 6:179. 10.1186/1754-6834-6-179 PMC3880215 24295562

[B95] Woo J. E. Lee S. Y. Jang Y.-S. (2018). Effects of nutritional enrichment on acid production from degenerated (non-solventogenic) *Clostridium acetobutylicum* strain M5. *Appl. Biol. Chem.* 61 469–472. 10.1007/s13765-018-0372-6

[B96] Woolston B. M. Emerson D. F. Currie D. H. Stephanopoulos G. (2018). Rediverting carbon flux in *Clostridium ljungdahlii* using CRISPR interference (CRISPRi). *Metab. Eng.* 48 243–253. 10.1016/j.ymben.2018.06.006 29906505

[B97] Xin F. Yan W. Zhou J. Wu H. Dong W. Ma J. (2018). Exploitation of novel wild type solventogenic strains for butanol production. *Biotechnol. Biofuels* 11:252. 10.1186/s13068-018-1252-3 PMC6145368 30250504

[B98] Xu T. Li Y. He Z. Van Nostrand J. D. Zhou J. (2017). Cas9 nickase-assisted RNA repression enables stable and efficient manipulation of essential metabolic genes in *Clostridium cellulolyticum*. *Front. Microbiol.* 8:1744 10.3389/fmicb.2017.01744 PMC5594222 28936208

[B99] Xu T. Li Y. Shi Z. Hemme C. L. Li Y. Zhu Y. (2015). Efficient genome editing in *Clostridium cellulolyticum* via CRISPR-Cas9 nickase. *Appl. Environ. Microbiol.* 81 4423–4431. 10.1128/aem.00873-15 25911483 PMC4475897

[B100] Yamano T. Nishimasu H. Zetsche B. Hirano H. Slaymaker I. M. Li Y. (2016). Crystal structure of Cpf1 in complex with guide RNA and target DNA. *Cell* 165 949–962. 27114038 10.1016/j.cell.2016.04.003 PMC4899970

[B101] Yoo S. M. Na D. Lee S. Y. (2013). Design and use of synthetic regulatory small RNAs to control gene expression in *Escherichia coli*. *Nat. Protoc.* 8 1694–1707. 10.1038/nprot.2013.105 23928502

[B102] Zetsche B. Gootenberg J. S. Abudayyeh O. O. Slaymaker I. M. Makarova K. S. Essletzbichler P. (2015). Cpf1 is a single RNA-guided endonuclease of a class 2 CRISPR-Cas system. *Cell* 163 759–771. 10.1016/j.cell.2015.09.038 26422227 PMC4638220

[B103] Zetsche B. Heidenreich M. Mohanraju P. Fedorova I. Kneppers J. DeGennaro E. M. (2017). Multiplex gene editing by CRISPR-Cpf1 using a single crRNA array. *Nat. Biotechnol.* 35 31–34. 10.1038/nbt.3737 27918548 PMC5225075

[B104] Zhang J. Hong W. Zong W. Wang P. Wang Y. (2018a). Markerless genome editing in *Clostridium beijerinckii* using the CRISPR-Cpf1 system. *J. Biotechnol.* 284 27–30. 10.1016/j.jbiotec.2018.07.040 30081040

[B105] Zhang J. Zong W. Hong W. Zhang Z. T. Wang Y. (2018b). Exploiting endogenous CRISPR-Cas system for multiplex genome editing in *Clostridium tyrobutyricum* and engineer the strain for high-level butanol production. *Metab. Eng.* 47 49–59. 10.1016/j.ymben.2018.03.007 29530750

[B106] Zhang J. Liu Y. J. Cui G. Z. Cui Q. (2015). A novel arabinose-inducible genetic operation system developed for *Clostridium cellulolyticum*. *Biotechnol. Biofuels* 8:36 10.1186/s13068-015-0214-2 PMC4355141 25763107

[B107] Zhang N. Shao L. Jiang Y. Gu Y. Li Q. Liu J. (2015). I-SceI-mediated scarless gene modification via allelic exchange in *Clostridium*. *J. Microbiol. Methods* 108 49–60. 10.1016/j.mimet.2014.11.004 25451462

[B108] Zhang Y. Xu S. Chai C. Yang S. Jiang W. Minton N. P. (2016). Development of an inducible transposon system for efficient random mutagenesis in *Clostridium acetobutylicum*. *FEMS Microbiol. Lett.* 363:fnw065 10.1093/femsle/fnw065 PMC4941238 27001972

[B109] Zhao J. P. Zhu H. Guo X. P. Sun Y. C. (2018). AU-rich long 3’ untranslated region regulates gene expression in bacteria. *Front. Microbiol.* 9:3080 10.3389/fmicb.2018.03080 PMC6299119 30619162

[B110] Zhao R. Liu Y. Zhang H. Chai C. Wang J. Jiang W. (2019). CRISPR-cas12a-mediated gene deletion and regulation in *Clostridium ljungdahlii* and its application in carbon flux redirection in synthesis gas fermentation. *ACS Synth. Biol.* 8 2270–2279. 10.1021/acssynbio.9b00033 31526005

[B111] Zheng Y. Su T. Qi Q. (2019). Microbial CRISPRi and CRISPRa systems for metabolic engineering. *Biotechnol. Bioproc. Eng.* 24 579–591.

[B112] Zhong J. Karberg M. Lambowitz A. M. (2003). Targeted and random bacterial gene disruption using a group II intron (targetron) vector containing a retrotransposition-activated selectable marker. *Nucleic Acids Res.* 31 1656–1664. 10.1093/nar/gkg248 12626707 PMC152852

